# Inhibition of the Rumen Ciliate *Entodinium caudatum* by Antibiotics

**DOI:** 10.3389/fmicb.2017.01189

**Published:** 2017-06-28

**Authors:** Tansol Park, Tea Meulia, Jeffrey L. Firkins, Zhongtang Yu

**Affiliations:** ^1^Department of Animal Sciences, The Ohio State UniversityColumbus, OH, United States; ^2^Molecular and Cellular Imaging Center, Ohio Agricultural Research and Development Center and the Department of Plant Pathology, Ohio State UniversityWooster, OH, United States

**Keywords:** antibiotics, associated bacteria, axenic culture, *Entodinium*, ruminal protozoa

## Abstract

Axenic cultures of free-living aerobic ciliates, such as *Tetrahymena thermophila* and *Paramecium aurelia*, have been established and routinely used in laboratory research, greatly facilitating, or enabling characterization of their metabolism, physiology, and ecology. Ruminal protozoa are anaerobic ciliates, and they play important roles in feed digestion and fermentation. Although, repeatedly attempted, no laboratory-maintainable axenic culture of ruminal ciliates has been established. When axenic ciliate cultures are developed, antibiotics are required to eliminate the accompanying bacteria. Ruminal ciliates gradually lose viability upon antibiotic treatments, and the resultant axenic cultures can only last for short periods of time. The objective of this study was to evaluate eight antibiotics that have been evaluated in developing axenic cultures of ruminal ciliates, for their toxicity to *Entodinium caudatum*, which is the most predominant ruminal ciliate species. Scanning and transmission electron microscopy (TEM) showed that the antibiotics damaged both the cell surface and nuclei of *E. caudatum* and increased accumulation of intracellular glycogen. Combinations of the three least toxic antibiotics failed to eliminate the bacteria that are present in the *E. caudatum* culture. The combination of ampicillin, carbenicillin, streptomycin, and oxytetracycline was able to eliminate all the bacteria, but the resultant axenic *E. caudatum* culture gradually lost viability. Adding the bacterial fraction (live) separated from an untreated *E. caudatum* culture reversed the viability decline and recovered the growth of the treated *E. caudatum* culture, whereas feeding nine strains of live bacteria isolated from *E. caudatum* cells, either individually or in combination, could not. Nutritional and metabolic dependence on its associated bacteria, accompanied with direct and indirect inhibition by antibiotics, makes it difficult to establish an axenic culture of *E. caudatum*. Monoxenic or polyxenic cultures of *E. caudatum* could be developed if the essential symbiotic partner(s) can be identified.

## Introduction

Pure cultures of microorganisms had been a gold standard for microbiological experimentation until the end of the twentieth century, and most of the current knowledge on microbial metabolism, physiology, and ecology were obtained from studies using pure cultures of microorganisms (Schmidt, 2006). Although, the contemporary ~omic technologies allow direct studies of microbes, pure cultures still enable, or at least facilitate, elucidation of the physiology and metabolism expressed in the niche of individual microbes. Therefore, the initial effort in traditional microbiological research is to obtain pure cultures for detailed studies. Moreover, pure cultures are needed to utilize the unique metabolic capacities of some microbes to produce compounds and products of commercial interest. In protozoology research, the early effort was made and succeeded in establishing axenic cultures of model protozoan species (Lwoff, 1923; Biagini et al., 1998). Because of their large cell sizes, protozoa can be isolated and maintained as cultures of single protozoal species by picking individual protozoal cells under a microscope. These single-species protozoal cultures contain bacteria and archaea, and combinations of antibiotics are used to kill off the contaminating bacteria and archaea. *Tetrahymena thermophila*, which is an aerobic free-living ciliate residing in freshwater and used as a model species of ciliates, was the first axenically grown protozoal species (Lwoff, 1923). Axenic cultures of *Paramecium aurelia*, another free-living freshwater model ciliate species, was also established (Soldo, 1963). Then, axenic cultures of several parasitic ciliates, including *Hexamita* (Biagini et al., 1998) and *Giardia ardeae* (Erlandsen et al., 1990) and free-living marine ciliates (Yamada et al., 1997; Cho et al., 2002; Wilkens and Maas, 2012) were successfully established. The axenic cultures of these ciliated can be maintained in laboratory, and they have greatly facilitated or enabled characterization of their metabolism, physiology, and ecology. Although physical separation using migration, filtration, and centrifugation is helpful in establishing axenic cultures of protozoa, antibiotics (often a combination of several antibiotics) are indispensable to eliminate the bacteria and archaea that are present in the monocultures of protozoa (Allen and Nerad, 1978). Antibiotics are also added to media to decontaminate axenic cultures of protozoa and mammalian cells that are contaminated with microbes. The growth of the aforementioned axenic protozoa can be maintained by providing growth factors in the media. For *T. thermophila*, media and axenic stock cultures are commercially available, which has greatly facilitated and enabled studies of this species.

Ruminal protozoa comprise anaerobic ciliates, and they have been estimated to provide up to 50% of the rumen microbial biomass (Newbold et al., 2015). Ruminal ciliates contribute to feed degradation but also lower nitrogen utilization efficiency and increase methane production (Firkins et al., 2007; Newbold et al., 2015). Sitting at the top of the food chain in the rumen, ruminal ciliates prey on other microbes, primarily ruminal bacteria (Williams and Coleman, 1992). Through digesting the engulfed preys, these ciliates meet their nutrient requirements for growth factors, such as amino acids, nucleic acid, and vitamins, and other nutrients (Bonhomme, 1990). Some bacteria and archaea are also shown to be associated with ruminal ciliates (Lloyd et al., 1996), but it remains unknown if these associated prokaryotes (endo-or ecto-symbiotic) provide any growth factors that are absolutely needed for the survival of ruminal protozoa. To better understand ruminal ciliates, many researchers have attempted to develop axenic cultures of ruminal ciliate protozoal species, especially *E. caudatum*, the most dominant ruminal ciliate species in ruminant animals (Sylvester et al., 2005). However, none of the attempts has succeeded in developing any axenic culture of ruminal ciliates in the laboratory that could be maintained for long-term use (Coleman, 1962; Hino and Kametaka, 1977; Bonhomme et al., 1982a,b). We hypothesize that the failure to establish and maintain axenic cultures of ruminal ciliates is probably attributable to the toxicity of antibiotics to ruminal protozoa. Toxicity of antibiotics to eukaryotes has been discussed for a long time, mainly in the context of clinical applications such as side effects or antibiotic resistance and, or free-living unicellular as model organisms (Eustice and Wilhelm, 1984; Van Bambeke et al., 2003; Barnhill et al., 2012). However, few studies in the literature have systematically addressed potential toxicity of antibiotics to ruminal ciliates (Varadyova et al., 2001). The objective of the present study was to investigate possible toxicity of eight antibiotics, which have been used in attempts to make axenic cultures of various types of ciliates including *E. caudatum*. Both scanning electron microscopy (SEM) and transmission electron microscopy (TEM) were used to reveal the morphological changes of *E. caudatum* cells caused by the antibiotics.

## Materials and methods

### *E. caudatum* culture

The *E. caudatum* culture used in the present study was initially established from a single cell isolated from the rumen of a gerenuk by Dehority (2010). This culture contained only *E. caudatum* as ruminal ciliates, but it also contained bacteria and probably archaea. This *E. caudatum* culture has been maintained by frequent transfers (50% inoculum) every 3–4 days into autoclaved SP medium (Dehority, 1998), and it is also cryopreserved at −80°C in the presence of 4% (v/v) dimethylsulphoxide as the cryoprotectant (Nsabimana et al., 2003). A protozoan feed consisting of ground wheat grain and alfalfa and grass hays was fed daily as recommend by Dehority (2010). The volume of the culture was increased by adding fresh SP medium, which contained (per liter) 5 g of K_2_HPO_4_, 4 g of KH_2_PO_4_, 1 g of NaCl, 0.053 g of CaCl_2_·2H_2_O, 0.0385 g of MgSO_4_, 6 g of NaHCO_3_, 10% (v/v) of clarified rumen fluid, 0.67% (v/v) of 3% cysteine-HCl solution to the culture to prepare adequate *E. caudatum* cells needed for the large number of antibiotic treatments.

### Antibiotics

Eight different antibiotics (Table 1) were tested for their efficacy to test their toxicity to *E. caudatum* cells and to eliminate the bacteria present in the *E. caudatum* culture. A stock solution of each antibiotic was prepared by dissolving in distilled water (except for chloramphenicol, which was dissolved in ethanol because it is insoluble in water) and filter-sterilized using Minisart syringe filters (pore size, 0.22 μm; Sartorius, Germany). The detailed information on concentrations used, target bacteria, and mechanism and mode of action is shown in Table 1. Dosages of each antibiotic were selected based on both references listed in Table 2 and the results of a preliminary experiment using the same *E. caudatum* culture.

**Table 1:** List of all antibiotics used in this study^*^.

**Antibiotic**	**Stock solution**	**Final Conc**.	**Main mechanism of action**	**Antimicrobial spectrum**	**Bactericidal/Bacteriostatic**
Ampicillin (Sigma, A9518)	50 mg/ml in H_2_O	0.1–2 mg/ml	Cell wall	Gram (+), (−)	Bactericidal
Streptomycin (Sigma, S9137)	25 mg/ml in H_2_O	0.1–2 mg/ml	Aminoglycoside, 30S protein synthesis	Gram (+), (−)	Bactericidal
Carbenicillin (Sigma, C3416)	50 mg/ml in H_2_O	0.1–2 mg/ml	Cell wall	Gram (+), (−)	Bactericidal
Tetracycline (Sigma, T3383)	50 mg/ml in H_2_O	0.03–0.3 mg/ml	30S protein synthesis	Gram (+), (−)	Bacteriostatic
Neomycin (Sigma, N6910)	50 mg/ml in H_2_O	0.1–1 mg/ml	Aminoglycoside, 30S protein synthesis	Gram (+), (−)	Bactericidal
Bacitracin (Sigma, B5150)	50 mg/ml in 1N HCl	0.05–1 mg/ml	Cell wall	Gram (+)	Bactericidal
Normocin™ (InvivoGen)	Commercial	0.05–0.5 mg/ml	50S protein synthesis + DNA synthesis + disrupting ionic exchange through cell membrane (yeast & fungi)	Gram (+), (−) Fungi	Bactericidal
Chloramphenicol (Sigma, C0378)	50 mg/ml in ethanol	0.005–0.05 mg/ml	50S protein synthesis	Gram (+), (−)	Bacteriostatic

* *Based on Gottlieb and Shaw, 1967; Foster, 1983; Moazed and Noller, 1987; Ocampo et al., 2014*.

**Table 2:** Literature review about previous research on establishing axenic culture of protozoa and algae.

**Host microbes**	**Bacterial contamination**	**Protozoal growth**	**Combination of antibiotics**	**References**
*E. caudatum*	Low bacterial contamination (10^3^–10^4^ bacteria/ml)	*Entodinia* could be maintained alive for only 3–4 days	Penicillin (1,400 U/ml) Streptomycin sulfate (570 μg/ml) Dihydrostreptomycin (570 μg/ml) Neomycin sulfate (570 μg/ml)	Coleman, 1962
*E. caudatum*	Enabled inhibition of growth completely; no growth was seen up to 10 days	These axenic protozoa could be kept alive in the presence of dead bacterial cells for up to 3 weeks, but their growth was extremely slow	Carbenicillin (500 μg/ml) Aminobenzylpenicillin (500 μg/ml) Cephaloridine (500 μg/ml) Chloramphenicol (100 μg/ml) Leucomycin (100 μg/ml)	Hino and Kametaka, 1977
Cellulolytic Flagellate *Trichomitopsis termopsidis* (from hindgut of termite)	Axenic status after 2 passages (in 30 days)	*T. termopsidis* did multiply w/10% (v/v) autoclaved rumen fluid + cellulose + GSH + serum, and antibiotics	Penicillin (1,000 U/ml) = 600 μg/ml Streptomycin (1 mg/ml)	Yamin, 1978
*Paramecium* spp.	Axenic culture was maintained (Migration + adaptation medium plus antibiotics)	The ciliates can be maintain in the growth medium	Penicillin (100 U/ml), Streptomycin (100 μg/ml), and fungizone (0.25 μg/ml)	Allen and Nerad, 1978
*E. caudatum*	Low bacterial contamination levels	Cell-free extract of mixed rumen bacteria adsorbed on activated charcoal was relieved the stress of low bacterial contamination	50 μg/ml each of streptomycin, penicillin and chloramphenicol and sulphadrug	Onodera and Henderson, 1980
*Giardia ardeae*	Axenic culture (confirmed through SEM)	Established in axenic culture using the TYI-S-33 medium	Bacitracin (50 μg/ml) Gentamicin (100 μg/ml) Penicillin (25 U/ml) Streptomycin (25 μg/ml) Amphotericin B (0.06 μg/ml)	Erlandsen et al., 1990
*E. exiguum* & *E. caudatum*	The antibiotic solution effectively killed more than 99% of the bacteria in 4 h (bacterial counts using MPN)	Much better growth with live bacteria	Approximately, Penicillin (1250 U/ml) Streptomycin (81.25 U/ml)	Fondevila and Dehority, 2001
Marine microalga *Isochrysis galbana*	Removal of bacteria was accomplished using a mixture of 5 antibiotics (axenic after 3 days incubation)	Similar growth with reference culture	Ampicillin (500 μg/ml) Gentamycin (100 μg/ml) Kanamycin (200 μg/ml) Neomycin (1 mg/ml) Streptomycin (100 μg/ml)	Cho et al., 2002
Rumen ciliates: *Polyplastron multivesiculatum, Isotricha intestinalis*, and *Ophryoscolex purkynjei*	No PCR amplification of bacterial or archaeal 16S rRNA gene from the culture supernatant (48 h incubation)	Over 90% was maintained after 48 h with antibiotics mixture	Penicillin G potassium (100 μg/ml) Streptomycin sulfate (100 μg/ml) Kanamycin sulfate (100 μg/ml) 5-Fluorocytosine (50 μg/ml) Chloramphenicol (3.2 μg/ml)	Irbis and Ushida, 2004
*Tetrahymena* spp.	Axenic culture	–	Neomycin (100 μg/ml), Kanamycin (100 μg/ml), Tetracycline (100 μg/ml) Normocin™(2 μl/ml) Normocin™, Penicillin (250 μg/ml), Streptomycin (250 μg/ml) Three-fold of Normocin™(6 μl/ml)	Cassidy-Hanley, 2012

### Experiment 1: growth inhibition of *E. caudatum* and its associated prokaryotes by individual antibiotics

#### Culturing

The *E. caudatum* culture was filtered and then washed three times as described previously using a filtration apparatus (Williams and Yarlett, 1982; Williams and Coleman, 1992) to remove most of the contaminating prokaryotes. Briefly, the *E. caudatum* culture was filtered (with washing) sequentially through 50- and then 25-μm nylon filter membranes (Sefar Filtration Inc., New York, USA) to remove the feed particles and then through a 10-μm nylon filter membrane that retains the *E. caudatum* cells but allows the free-living prokaryotes to pass through. Pre-warmed (at 39°C) anaerobic Simplex buffer (modified from Williams and Coleman, 1992), which was prepared anaerobically by adding 0.02% (w/v) of L-cysteine-hydrochloride and sparging with CO_2_ overnight, was used to wash the *E. caudatum* cells retained on the 10-μm nylon filter membrane. To protect the *E. caudatum* cells from exposure to air during the filtration, four continuous fluxes of CO_2_ were directed above the filter membrane through ports pointing down on the inside of the filtration apparatus (Williams and Yarlett, 1982). The washed *E. caudatum* cells were collected into pre-warmed (39°C) Simplex buffer of the same volume as the original culture. Suspensions of the washed *E. caudatum* cells (0.5 ml each) were inoculated into 4.5 ml of anaerobic SP medium (Dehority, 1998). Approximately 0.01 g of the protozoan feed was added to each tube. The cell concentration of the *E. caudatum* cell suspension was determined by microscopic counting (Dehority, 1993). Briefly, a small aliquot of the cell suspension was mixed with an equal volume each of 50% formalin and 30% glycerol. The fixed *E. caudatum* cells were loaded onto a Sedgewick-Rafter counting chamber (Thomas Scientific, no. 9851 C20, Swedesboro, NJ). The *E. caudatum* cells within 50 random 0.25 mm^2^ grids in the microscope eyepiece were counted twice under 100x total magnification. To aid microscopic counting, the fixed *E. caudatum* cells were stained with brilliant green dye.

Growth inhibition and toxicity of each antibiotic were evaluated using the suspension of the washed *E. caudatum* cells as the inoculum. Briefly, each of the selected antibiotics was added to the *E. caudatum* cultures at the pre-set final concentrations (Table 1). After flushing the headspace with filter-sterilized CO_2_, the culture tubes were sealed with a rubber stopper and incubated at 39°C at a 10° angle (Dehority, 1998). Each *E. caudatum* culture was subsampled (0.5 ml each time) at 24, 48, and 72 h, and the concentration of *E. caudatum* was determined using microscopic counting as described above. The growth of associated prokaryotes was estimated by optical density (OD) at 600 nm using a Spectronic 20D+ (Spectronic Instruments, USA) before each subsampling. Sterile SP medium was used as the blank to zero the spectrophotometer (the cells of *E. caudatum* settle to the bottom of the cultures and contribute little to culture OD).

#### Electron microscopy

*E. caudatum* cells were collected from all cultures (both the antibiotics-treated and the controls receiving no antibiotics) by centrifugation at 500 × g for 5 min. The cells were fixed in a fixative buffer, which contains 0.1 M potassium phosphate (pH 7.2), 3% glutaraldehyde, and 2% paraformaldehyde, for 2 h at room temperature and then stored at 4°C overnight. Samples were then divided into two aliquots with one for scanning and the other for TEM processing.

For SEM, cells were rinsed with a potassium phosphate buffer (PB, 0.1 M, pH 7.2) thrice, 10 min each. After each rinse and after each of the following steps, the cells were collected by centrifugation as mentioned above. Cells were then post-fixed in 1% osmium tetroxide and 1% uranyl acetate in PB for 1 h, washed twice with PB, and then dehydrated through sequential washes in 50, 75, 95% ethanol twice (15 min each at each ethanol concentration) and then in 100% ethanol twice (15 min each). Dehydrated cells were transferred to 100% hexamethyldisilazane (Sigma-Aldrich Ltd, St-Louis, USA). After two changes of the hexamethyldisilazane, the cells air-dried a chemical hood. Finally, the cells were mounted onto a specimen holder and subsequently spatter coated with platinum and viewed on a Hitachi S-4700 (Hitachi America, Ltd.) scanning electron microscope.

For TEM, fixed cells were collected by low-speed centrifugation at 500 × g for 5 min and embedded in 0.8% low melting agarose (Thermofisher Scientific Company, Waltham, MA). The cells/agarose blocks were washed thrice with PB and post-fixed with 1% osmium tetroxide and 1% uranyl acetate in PB at room temperature for 1 h. The cells were then dehydrated through a graded ethanol series (50, 75, 95, and 100%) as described above for SEM, resin-infiltrated using a propylene oxide-resin series, and then embedded in EM Bed-812 resin (Electron Microscopy Sciences, Hartfield, PA). Ultrathin-sections (60–70 nm) were prepared using a Leica EM UC6 ultra-microtome. Sections from five separate blocks were obtained for each sample. After staining with 3% aqueous uranyl acetate for 20 min, followed by Reynolds' lead citrate for 10 min (Reynolds, 1963), all the sections were viewed using a Hitachi H-7500 transmission electron microscope (Hitachi America, Ltd.). Images were acquired using an Optronics QuantiFire® S99835 (SIA) digital camera. Size (area) of the glycogen granules of the control and antibiotics-treated cell was measured using ImageJ (Abràmoff et al., 2004).

### Experiment 2: preparation of a temporarily axenic culture of *E. caudatum* and its growth recovery

Three of the antibiotics tested above (carbenicillin, bacitracin, and neomycin) were used in combinations to eliminate the contaminating bacteria from the *E. caudatum* culture in an attempt to develop an axenic culture of *E. caudatum*. These three antibiotics were chosen based on the results of Experiment 1 with respect to their low toxicity to *E. caudatum* and based on their complementary modes of antibiotic action. Three combinations of antibiotics were used: carbenicillin (1 mg/ml) plus bacitracin (0.05 mg/ml), carbenicillin plus neomycin (0.1 mg/ml), and carbenicillin plus bacitracin and neomycin. The antibiotic concentrations were selected based on their antibacterial activity and toxicity to the growth and morphology of *E. caudatum*. Soluble starch (0.1%, w/v), instead of the particulate protozoal feed, was added to SP medium as the substrate to support the growth of *E. caudatum* (because soluble starch is a better substrate than the particulate protozoal feed and the presence of the antibiotics prevents bacterial overgrowth on starch).

External growth factors were tested for their ability to support the growth of *E. caudatum*. The tested growth factors included fetal bovine serum (1%, v/v), stigmasterol (1 μg/ml), hemin (0.767 μM), and bovine serum albumin (0.005%, w/v), all of which have been used in developing axenic cultures of ciliate protozoa by other researchers (Yamin, 1978; Wagener and Pfennig, 1987; Schousboe et al., 1992; Mori et al., 2011). All growth factors were added at the concentrations recommended in the above studies or at concentrations slightly modified based on a preliminary study. Cultures receiving no growth factors were included as a control. The cultures were incubated at 39°C, and subsamples were collected at 0, 24, and 48 h. Following serial dilutions in the anaerobic Simplex buffer, 0.1 ml of each dilution was plated onto tryptic soy agar (TSA) plates prepared in an anaerobic chamber (Coy Laboratory Products Inc., USA) filled a gas mixture of 85% N_2_, 10% H_2_, and 5% CO_2_. After anaerobic incubation for 2–3 days at 39°C in the anaerobic chamber, bacterial colony forming units (CFU) were counted on each plate. *E. caudatum* cells in the subsamples were counted under a microscope as described in Experiment 1.

The three antibiotics used in Experiment 2 failed to eliminate the contaminating bacteria from the *E. caudatum* culture, so we tested another four antibiotics: ampicillin, carbenicillin, streptomycin, and oxytetracycline, all of which have been used in establishing axenic cultures of other ciliates. Briefly, a combination of ampicillin (1.0 mg/ml), streptomycin (0.2 mg/ml), carbenicillin (1 mg/ml), and oxytetracycline (0.2 mg/ml) was added to the cultures of *E. caudatum*. After incubation at 39°C for 3 days, the *E. caudatum* cells were counted under a microscope, while the elimination of the contaminating bacteria was confirmed by plating on TSA plates as described above. The temporarily axenic *E. caudatum* culture (based on confirmation of no CFU on the TSA plates) was washed once with SP medium to remove residual antibiotics and then transferred to sterile SP medium containing the protozoal feed that had been autoclaved in sealed tubes containing CO_2_. Cell suspensions of live bacteria (both individual and mixed) of the *E. caudatum* culture were tested for their ability to sustain the growth of the temporary axenic *E. caudatum* culture. Briefly, an actively growing *E. caudatum* culture that had not been treated with any antibiotic was filtered through a 10-μm filter membrane as described above. The filtrate containing only the prokaryotes was centrifuged at 4°C to pellet the bacterial (and possibly archaeal) cells. After washing once in the anaerobic Simplex buffer, the cells of the mixed prokaryotes were resuspended in anaerobic SP medium (in the same volume of the filtrate, referred to as mixed bacteria). The *E. caudatum* cells retained on the filter membrane were recovered and washed thrice using sterile anaerobic Simplex buffer as described above. The washed *E. caudatum* cells were lysed using low-speed bead-beating (2,400 oscillations/min) for 45 s using sterile zirconia beads (0.3 g of 0.1 mm and 0.1 g of 0.5 mm; Biospec Products, Bartlesville, OK), and the lysate was serially diluted in anaerobic Simplex buffer and plated on TSA plates to isolate bacteria associated with the *E. caudatum* cells. After incubation anaerobically at 39°C for 48 h, colonies that were well-separated and with distinctively different colony morphologies were inoculated into tryptic soy broth medium and incubated anaerobically at 39°C overnight. The cells from each bacterial isolate were harvested by centrifugation at room temperature, washed once in anaerobic Simplex buffer, and resuspended in SP medium (in the same volume of the original culture, referred to as individual bacterial isolates). Cell suspensions of nine individual bacterial isolates were prepared. Cell suspensions (1 ml each) of the individual bacterial isolates, combinations of three of the nine bacteria, and the mixed bacteria were fed individually to the temporarily axenic *E. caudatum* culture daily. The control received no live bacteria.

### Statistical analysis

All the cultures of *E. caudatum*, both antibiotics-treated and the control, were grown in three replicates. The *E. caudatum* counts in the antibiotics-treated cultures were expressed as % of that of the control culture that received no antibiotics, and culture OD (as an estimate of bacterial concentration) was subjected to two-way ANOVA using SAS 9.3 (SAS Institute, Cary, NC, USA). Orthogonal polynomial contrasts were used to determine linear or quadratic changes of protozoal cell counts by increasing concentrations of each antibiotic. Correlation between protozoal cell counts and OD of associated prokaryotes was examined using Pearson's correlation coefficient. Significant difference was declared at *P* ≤ 0.05.

## Results

### Experiment 1. growth inhibition of *E. caudatum* and its associated prokaryotes by antibiotics

All the antibiotic treatments significantly lowered the cell counts of *E. caudatum* except several of the antibiotics at the low concentrations, and the inhibition was aggravated at the later hours of the incubation (Table 3). Except for ampicillin, all the antibiotics exhibited a significantly linear dose-dependent inhibition of the growth of *E. caudatum* at 48 and 72 h incubation (Table 3). In the 72 h *E. caudatum* cultures containing 0.5 and 1 mg/ml of ampicillin, very few or no moving *E. caudatum* cells were seen under the microscope, but unexpectedly 2 mg/m ampicillin only lowered *E. caudatum* count by >54%. By the end of the 72 h incubation, protozoal cell counts did not differ between the control and the *E. caudatum* cultures receiving 0.1 or 0.5 mg/ml of streptomycin, 0.1 mg/ml of carbenicillin, or 0.1 mg/ml of neomycin even though these antibiotic-treated cultures had lower OD. Chloramphenicol exhibited the strongest inhibition of *E. caudatum* growth among the tested antibiotics, resulting in almost no live *E. caudatum* cells after 48 h incubation at all the tested concentrations.

**Table 3:** *E. caudatum* data (% of that of the control) in the presence of different antibiotics (mg/ml).

**Cell counts**	**Incubation times (h)**			**SEM**	**Effects**		
	**24**	**48**	**72**		**A**	**Time**	A^*^T
**Control**	12080^a^	19395^a^	19999^a^	1440			
**AMPICILLIN**							
0.1	0.83	0.47^ab^	0.52^ab^	0.12	<0.0001	<0.0001	0.0066
0.5	0.84	0.01^b^	0.01^b^	0.14			
1	0.83	0.43^ab^	0^b^	0.13			
2	0.85	0.63^a^	0.47^ab^	0.06			
**Contrast**	–	Q^***^	L^*^,Q^***^				
**STREPTOMYCIN**							
0.1	0.82	1.03^a^	1.11^a^	0.07	<0.0001	0.257	0.0031
0.5	0.95	0.89^a^	0.98a^b^	0.03			
1	0.69	0.55^b^	0.76^b^	0.05			
2	0.88	0.49^b^	0.42^*c*^	0.08			
**Contrast**	–	L^***^,Q^*^	L^***^				
**CARBENICILLIN**							
0.1	0.83	0.64^b^	0.85^a^	0.04	<0.0001	<0.0001	0.0004
0.5	0.87	0.45^b^	0.43^b^	0.07			
1	0.93	0.56^b^	0.44^b^	0.08			
2	0.78	0.49^b^	0.28^b^	0.08			
Contrast	-	L^***^,Q^***^	L^***^,Q^***^				
**TETRACYCLINE**							
0.03	0.94	0.58^b^	0.2^b^	0.11	<0.0001	<0.0001	0.0001
0.05	0.87	0.49^bc^	0.23^b^	0.1			
0.1	0.88	0.55^b^	0.17^b^	0.11			
0.3	0.81	0.24^c^	0.03^b^	0.12			
Contrast	-	L^***^,Q^**^	L^***^,Q^***^				
**NEOMYCIN**							
0.1	0.85	0.75^b^	0.96^a^	0.04	<0.0001	<0.0001	0.0011
0.3	0.84	0.53^bc^	0.51^b^	0.06			
0.6	0.76	0.51^c^	0.37^b^	0.06			
1	0.74	0.42^c^	0.33^b^	0.07			
Contrast	L^*^	L^***^,Q^***^	L^***^,Q^***^				
**BACITRACIN**							
0.05	0.89^ab^	0.72^ab^	0.56^b^	0.07	<0.0001	<0.0001	0.0514
0.1	0.93^ab^	0.58^b^	0.5^b^	0.08			
0.5	0.71^ab^	0.54^b^	0.4^b^	0.05			
1	0.49^b^	0.07^c^	0.01^c^	0.08			
Contrast	L^***^	L^***^	L^***^				
**NORMOCIN**™							
0.05	0.89^ab^	0.65^b^	0.56^b^	0.06	<0.0001	<0.0001	0.2012
0.1	0.69^ab^	0.47^bc^	0.57^b^	0.04			
0.3	0.69^ab^	0.39^bc^	0.31^c^	0.07			
0.5	0.5^b^	0.18^c^	0.1^c^	0.08			
Contrast	L^***^	L^***^,Q^**^	L^***^,Q^**^				
**CHLORAMPHENICOL**							
0.005	0.82^a^	0.11^b^	0.08^b^	0.13	<0.0001	<0.0001	<0.0001
0.01	0.76^ab^	0^b^	0^b^	0.13			
0.03	0.71^ab^	0^b^	0^b^	0.12			
0.05	0.39^b^	0^b^	0^b^	0.07			
**Contrast**	L^***^	L^***^,Q^***^	L^***^,Q^***^				

The final concentrations of antibiotics were in mg/ml. Different superscripts (a–c) in the same column denote significant difference (P < 0.05) in the proportion of protozoa cell counts between the control and each antibiotic treatment. Significance (

*** , P ≤ 0.01;

** , P ≤ 0.05;

* *, P ≤ 0.10) of linear (L) and quadratic (Q) contrast are shown. Each value was the mean of three replicates. A, antibiotics; T, incubation time*.

All the antibiotics (except bacitracin) at the tested concentrations also significantly lowered the culture OD, which was used as an indicator of cell concentration of the prokaryotes in the *E. caudatum* cultures (Figure S1). Different antibiotics lowered OD to different extent, and greater OD inhibition was seen for all the antibiotics except bacitracin. For streptomycin (at 0.1 and 2 mg/ml only), carbenicillin, tetracycline, neomycin (at 0.1 and 0.3 mg/ml only), Normocin™, bacitracin (at 0.1 and 1 mg/ml only), and chloramphenicol, prolonged incubation did not result in further OD decrease. Overall, the OD of the cultures receiving ampicillin increased (*P* = 0.003) significantly over time, while that of the other antibiotic-treated cultures did not. Except for streptomycin, all the tested antibiotics resulted in a correlation between the cell counts of *E. caudatum* and the OD (Table 4).

**Table 4:** Correlation between protozoal cell counts and optical density (OD) of antibiotics-treated *E. caudatum* cultures (*n* = 3).

**Antibiotics**	**Pearson**	***P*-value**
Overall	0.14	0.0167
Ampicillin	0.30	0.0421
Streptomycin	−0.11	0.4752
Carbenicillin	0.66	<0.0001
Tetracycline	0.62	<0.0001
Neomycin	0.64	<0.0001
Bacitracin	−0.51	0.0003
Normocin™	0.57	<0.0001
Chloramphenicol	0.80	<0.0001

**Figure 1:**
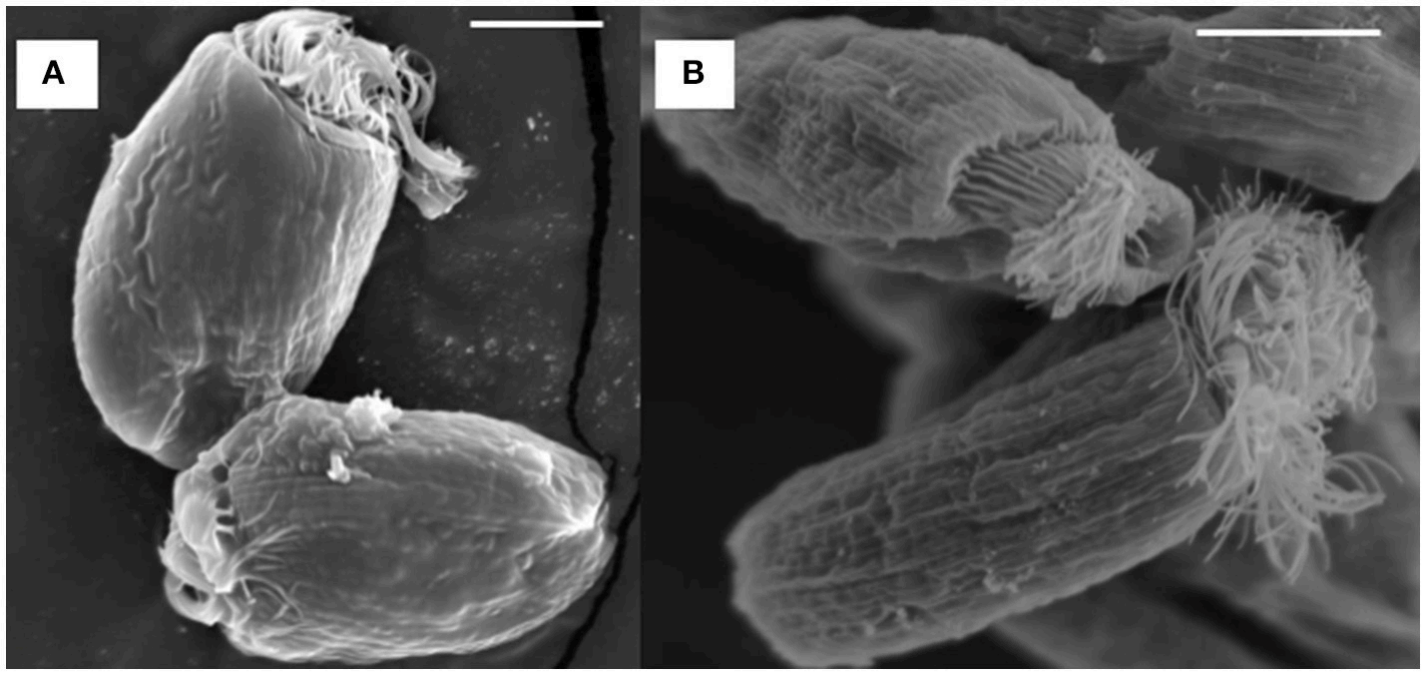
Scanning electron micrograph of *E. caudatum* cells maintained in the *in vitro* cultures before **(A)** and after **(B)** filtration and washing. The *in vitro* cultures did not receive any antibiotics. The scale bars = 10 μm.

**Figure 2:**
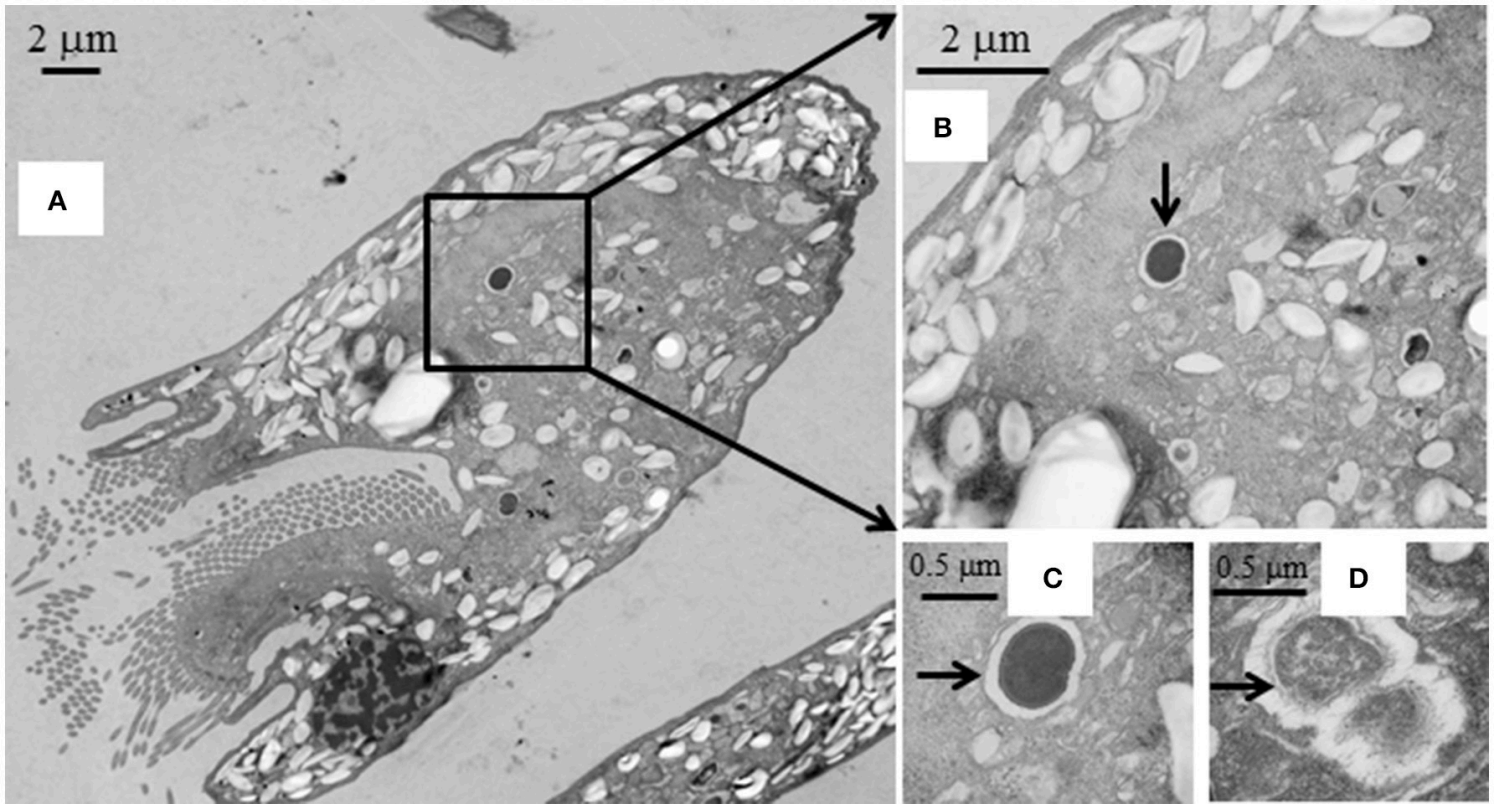
Transmission electron micrographs of *E. caudatum* cells that were not exposed to antibiotics. **(A)**, longitudinal section; **(B)**, a dividing microbial cell in the endoplasm (pointed by the arrow); **(C)**, amplified view of the dividing microbe; and **(D)**, a microbial cell being degraded/digested.

SEM showed that both unwashed and washed *E. caudatum* cells of the control culture had almost no prokaryotic microbes attached to their surface (Figure 1). No apparent damage to the extracellular morphology or structure was noted after the filtration or washing steps, suggesting that the filtration and washing procedures did not result in visible morphological damage to the *E. caudatum* cells. When examined using TEM, very few intact prokaryotic cells were visible in the cytoplasm of the cells of the washed *E. caudatum* cultures that did not receive any antibiotic (Figure 2). Dividing intracellular prokaryotic cells were observed inside vacuoles surrounded by a membrane (Figures 2B,C), whereas others appeared partially degraded (Figure 2D). No intracellular prokaryotic cell was visible on the TEM micrographs of the *E. caudatum* cells treated with carbenicillin or Normocin™, but one or less was seen on each section of the *E. caudatum* cells in the control culture (Table S1). Compared to the *E. caudatum* cells of the control culture, the antibiotics-treated *E. caudatum* cells appeared to have fewer dividing intracellular prokaryotes (Table S1).

**Figure 3:**
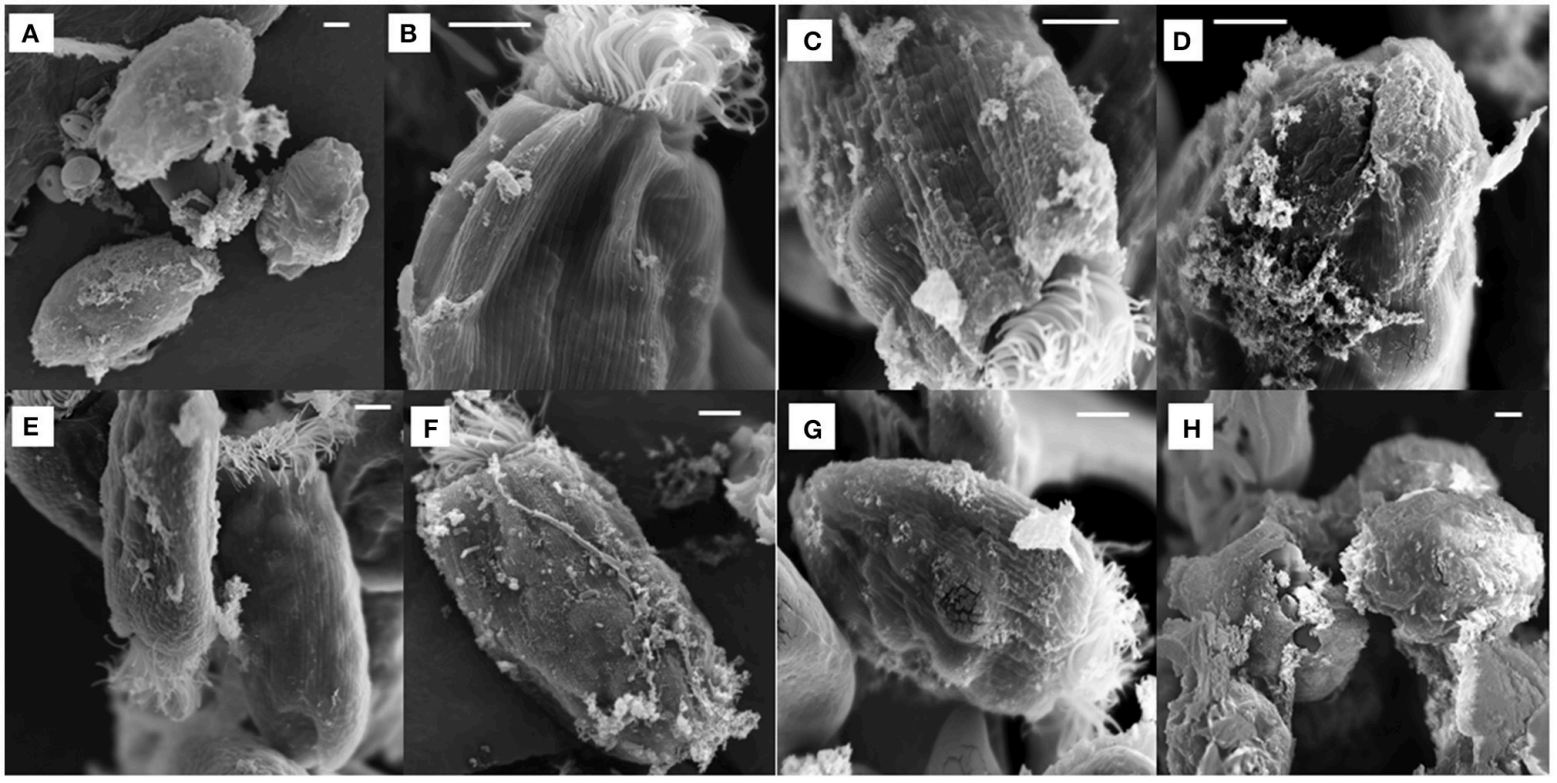
Scanning electron micrograph of *E. caudatum* cells in the antibiotics-treated cultures (after 48 h incubation). The micrograph of non-treated *E. caudatum* cells was shown in Figure 1. **(A)** ampicillin (0.5 mg/ml); **(B)** streptomycin (2 mg/ml); **(C)** carbenicillin (2 mg/ml); **(D)** tetracycline (0.3 mg/ml); **(E)**, neomycin (1 mg/ml); **(F)** bacitracin (1 mg/ml); **(G)** Normocin™ (0.5 mg/ml); and **(H)** chloramphenicol (0.005 mg/ml). The scale bars = 5 μm.

All the antibiotics-treated *E. caudatum* cells showed alteration or destruction of extracellular morphology as revealed on SEM micrographs (Figure 3), but it was impossible to quantify such changes. As revealed on SEM micrographs, ampicillin (Figure 3A), and chloramphenicol (Figure 3H) resulted in disrupted cell surface structures of the *E. caudatum* cells, with bulging and loss of longitudinal striation on the cell surface. Streptomycin (Figure 3B), tetracycline (Figure 3D), and Normocin™ (Figure 3G) also resulted in some distortion in the cuticle including swollen or shrunken pellicle structure. The ciliary zone was rather normal of the streptomycin-, carbenicillin-, neomycin-, and bacitracin-treated *E. caudatum* cells, whereas the other antibiotics totally destructed the ciliary zone. Bacitracin-treated *E. caudatum* cells had more surface-attached prokaryotic microbial cells (both rod-shaped and coccus cells in company with high culture OD) than those of the control and the other treatments.

**Figure 4:**
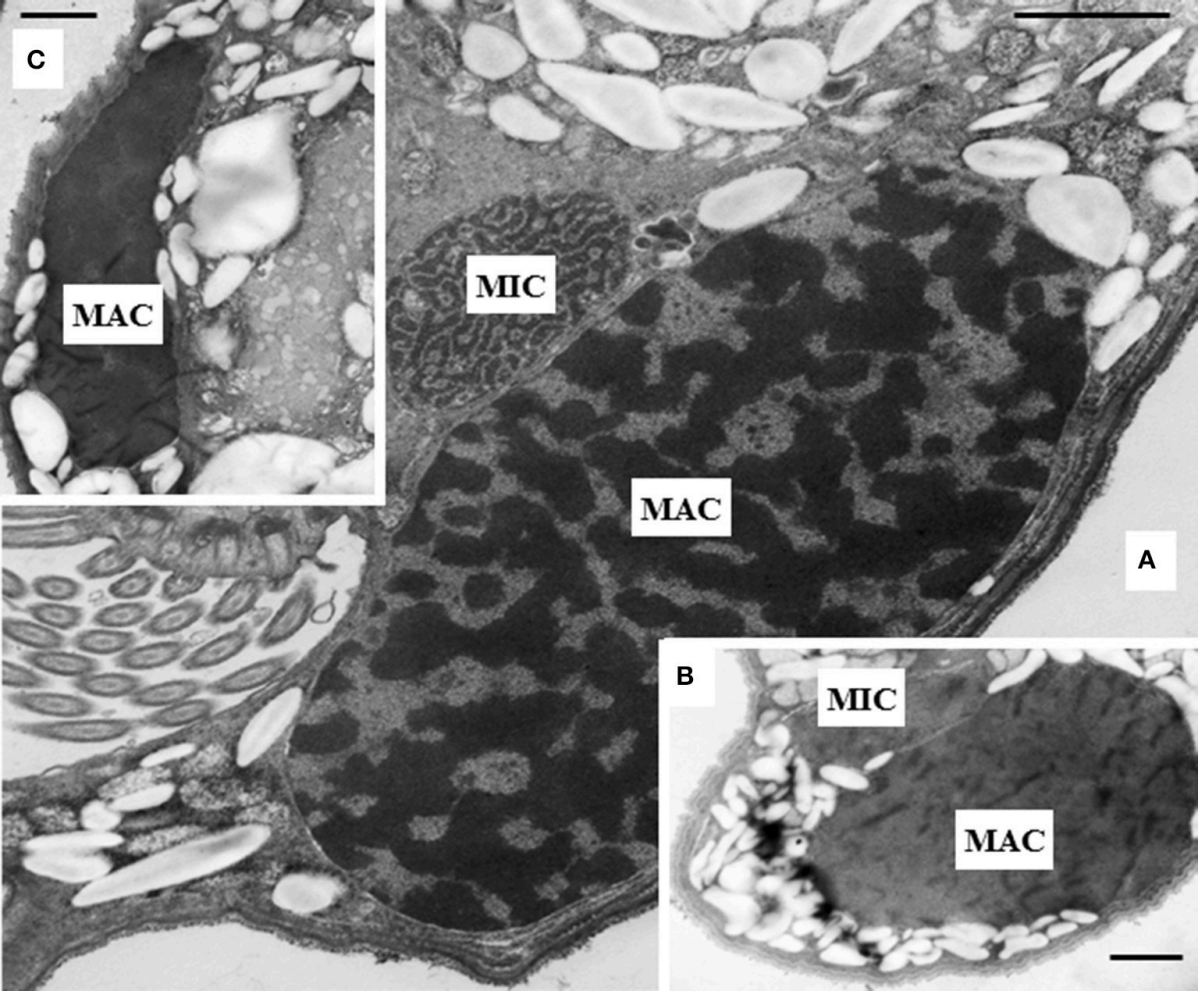
**(A)** Micronucleus (MIC) and macronucleus (MAC) in the ectoplasm of an *E*. *caudatum* cell that was not treated with any antibiotics. Both chromatin (electron dense dark areas) and granular nucleoli (gray areas) were visible in both the MIC and MAC. MAC and increased number of glycogen granules upon treatment with 1 mg/ml carbenicillin **(B)** and 0.1 mg/ml Normocin™ **(C)** for 48 h. The scale bars = 1 μm.

**Figure 5:**
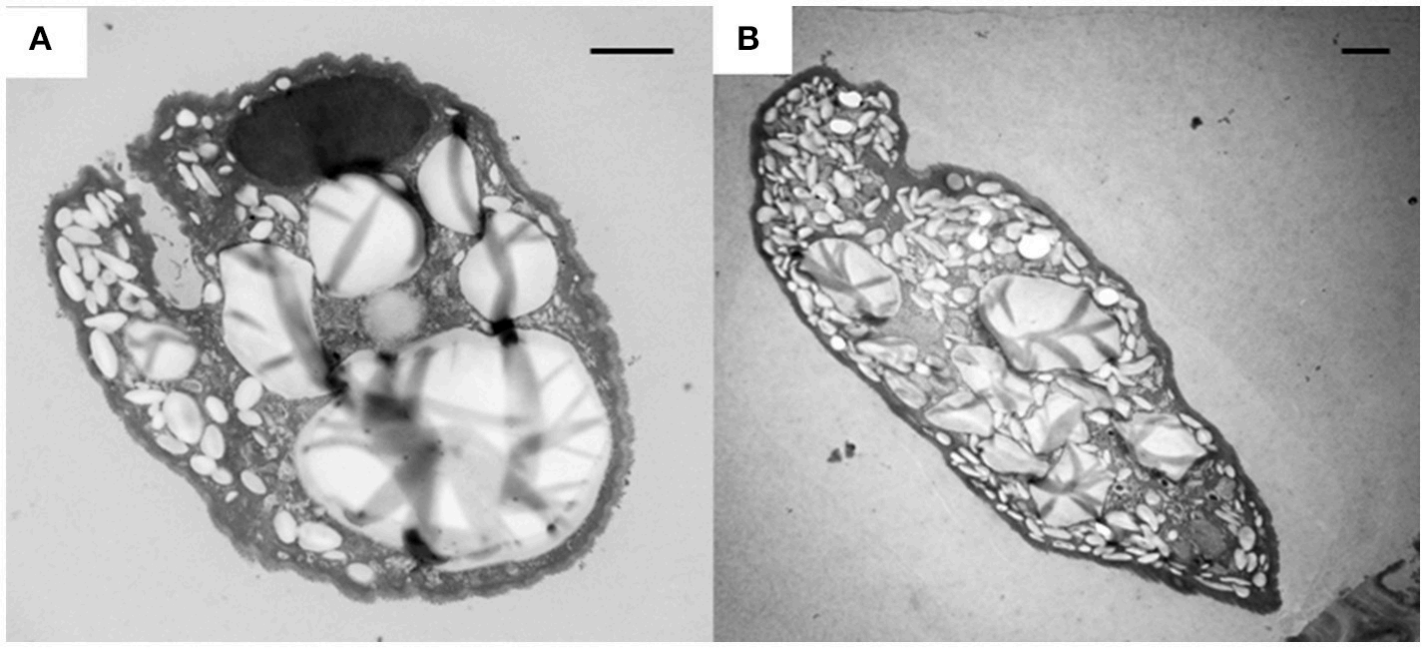
Accumulation of glycogen granules (electron lucent bodies) in *E. caudatum* cells treated with carbenicillin (1 mg/ml) for 24 h **(A)** and Normocin™ (0.1 mg/ml) for 48 h **(B)**. The scale bars = 2 μm.

**Figure 6:**
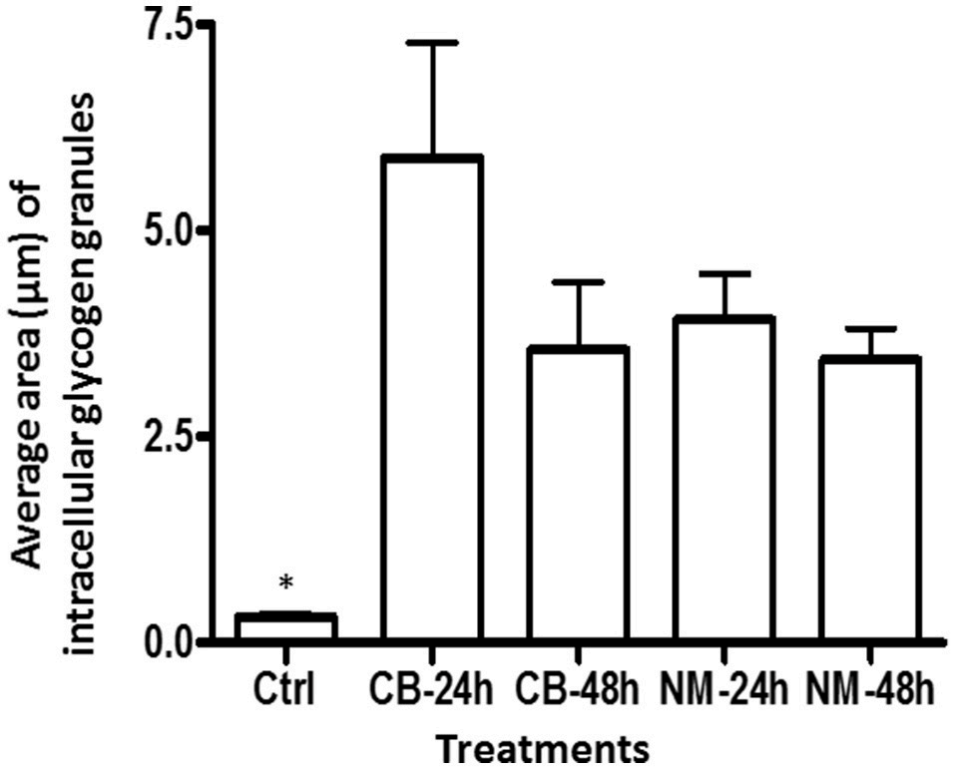
The average area of glycogen granules per longitudinally sectioned *E. caudatum* cell. Five largest granules were selected from each treatment and measured. Ctrl, untreated *E. caudatum* cells; CB, carbenicillin (1 mg/ml) treated cells; NM, Normocin™ (0.1 mg/ml) treated cells. The treatment cultures were incubated in the presence of each antibiotic for 24 or 48 h, while the control culture received no antibiotics and was incubated for 48 h. Error bars represent SEM (*n* = 5). ^*^, significant difference (*P* < 0.05) between the control and the treatments, but not among the treatments

**Figure 7:**
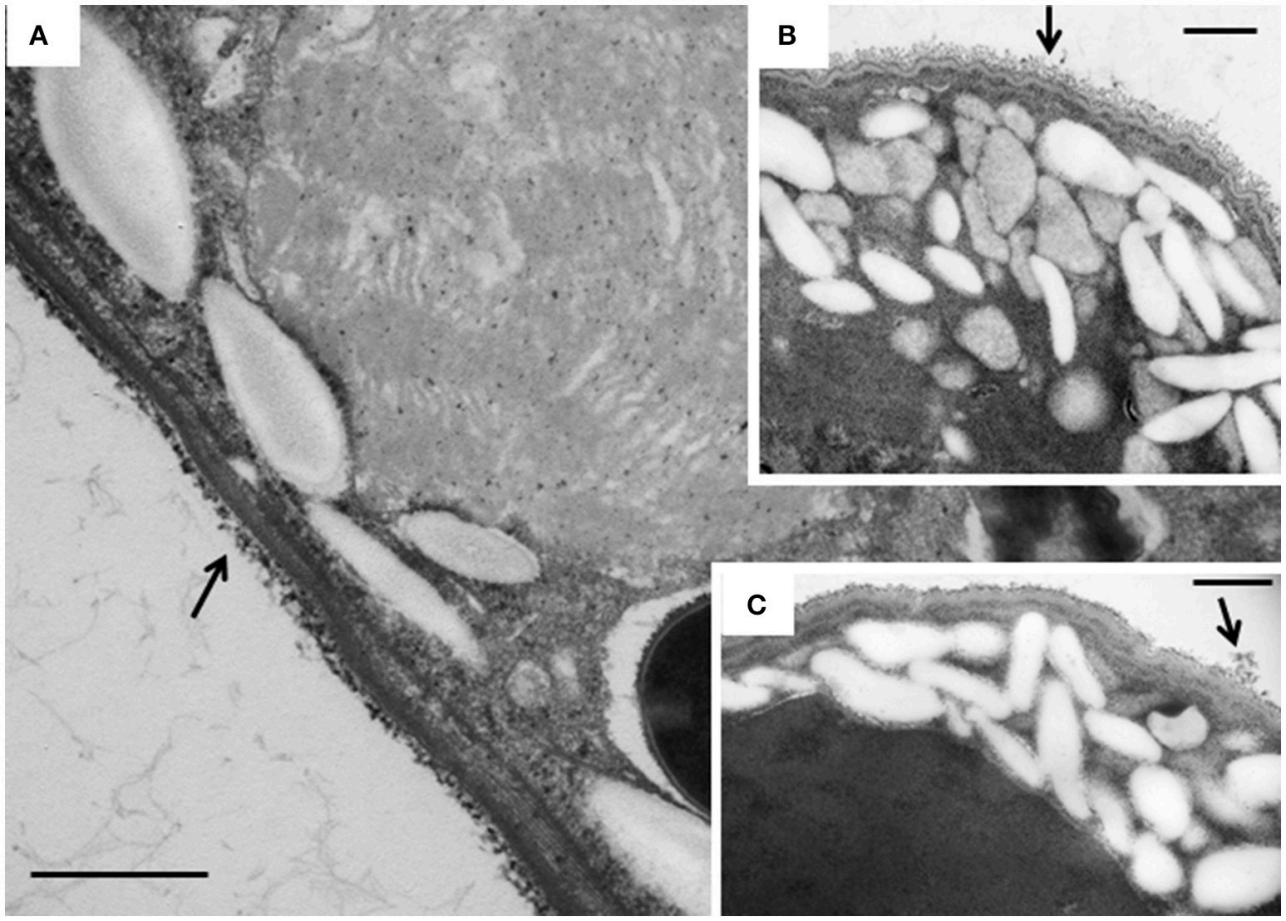
TEM micrographs of the cortex of *E. caudatum* cells. **(A)** untreated *E. caudatum* cells; **(B)** carbenicillin (1 mg/ml, for 48 h) treated cells; **(C)** Normocin™ (0.1 mg/ml, 24 h) treated cells. External filamentous glycocalyx (arrows) covering the cortical layers of *E. caudatum* cells showed morphological disparity between antibiotics-treated and untreated cells. The scale bars = 500 nm.

The intracellular structure of the *E. caudatum* cells was examined using TEM. Only the cells collected from the *E. caudatum* cultures treated with carbenicillin and Normocin™ were subjected to TEM. The *E. caudatum* cells treated by these two antibiotics were chosen because carbenicillin was one of the least toxic antibiotics (Figure 3, Table 3), and Normocin™ is commonly used in preventing mammalian cell cultures from bacterial contamination or decontaminating mammalian cell cultures and thus can be potentially used in developing axenic *E. caudatum* cultures. The *E. caudatum* cells from the control culture exhibited a clear distinction between the dense chromatin and granular nucleoli in both the MAC and MIC (Figure 4A), whereas all the *E. caudatum* cells in the cultures treated with either antibiotic lost such distinction in both the MIC and MAC (Figures 4B,C). No apparent difference in the degree of the damage, which was not possible to quantify, was noted between 24 h and for 48 h of exposure to these two antibiotics. Compared to the control, the exposure to these two antibiotics increased the number and the size of polysaccharide granules in all the observed cells (Table S1), which were mainly visualized in the ectoplasm as electron lucent bodies (Figure 5). When the areas of the largest 5 granules in 5 longitudinally sectioned *E. caudatum* cells were measured, carbenicillin and Normocin™ significantly (*P* < 0.05) increased the granule sizes compared to the control (Figure 6). Iodine staining of the *E. caudatum* cells showed that these granules are glycogen granules. In addition, sloughing of the glycocalyx covering the external membrane was noted, and electro-dense epiplasm, which consists of two underlying layers with occasional granularity underneath cell membrane, was not distinct inside the antibiotics treated cells (Figure 7). However, it was not possible to quantify the degree of such changes.

### Experiment 2: preparation of a temporarily axenic culture of *E. caudatum* and its growth recovery

**Figure 8:**
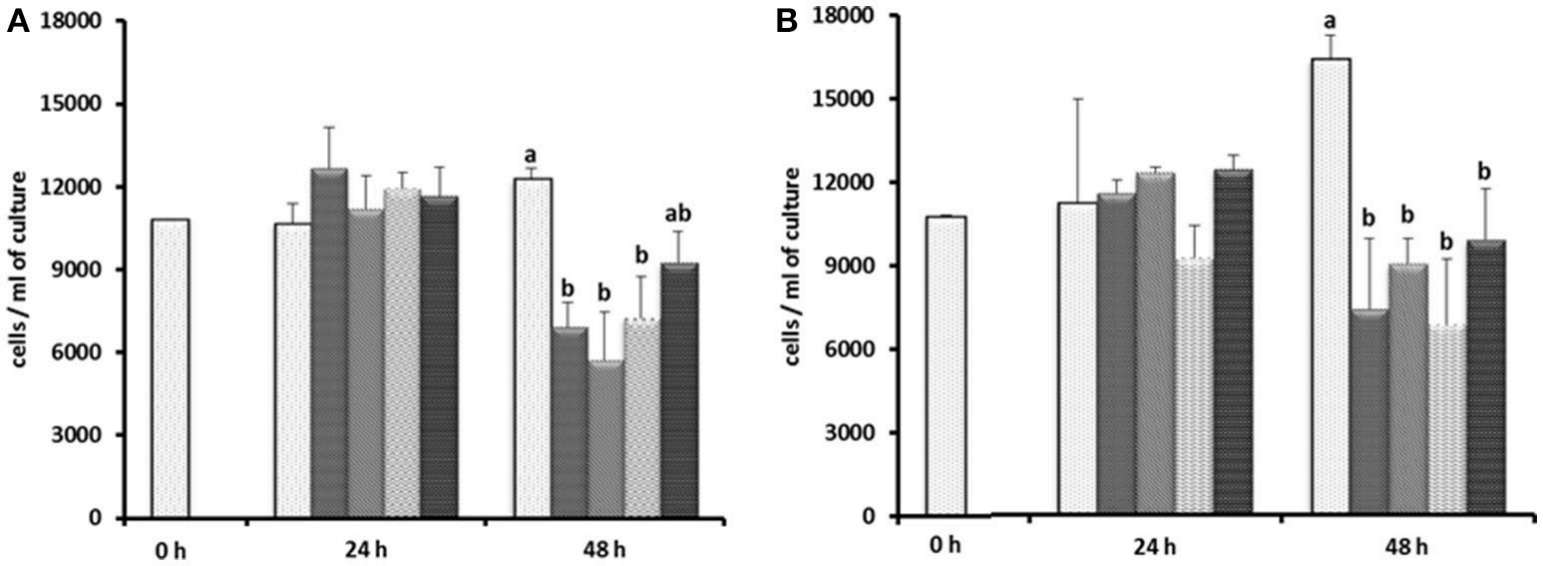
Effect of carbenicillin alone and its combination with bacitracin and neomycin and growth factors on *E. caudatum* cell counts. 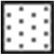 = control without any antibiotics; 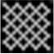 = carbenicillin (1 mg/ml); 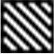 = combination of carbenicillin and bacitracin (0.05 mg/ml); 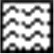 = combination of carbenicillin and neomycin (0.1 mg/ml); 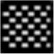 = combination of carbenicillin, bacitracin, and neomycin. **(A)**, no addition of growth factors; **(B)**, addition of 1% [v/v] fetal bovine serum, 1 μg/ml [w/v] stigmasterol, 0.767 μM heminm and 0.005% [w/v] bovine serum albumin. Erros bars SEM (*n* = 3).

Based on the results of Experiment 1, three of the antibiotics (carbenicillin, bacitracin, and neomycin) and their two- and three-way concentrations were used to generate an axenic culture of *E. caudatum*. These three antibiotics were chosen for three reasons. First, carbenicillin was one of the most effective antibiotics in inhibiting the prokaryotes in the *E. caudatum* culture (Figure S1) and the least toxic to the *E. caudatum* cells in terms of cellular surface damage (Figure 3) and decrease in viability (Table 3). Second, bacitracin is less toxic than the other antibiotics as revealed by the clear cytoplasmic matrix and a lack of increased accumulation of intracellular polysaccharide granules under a light microscope (data not shown). Third, neomycin has a broad spectrum of antibiotic activity by directly inhibiting protein synthesis in bacteria. The growth of *E. caudatum* was inhibited in the presence of the three antibiotics, alone and in combinations, irrespective the presence of the growth factors (Figure 8). The cultures containing growth factors only had numerically more *E. caudatum* cells than the control that received no growth factors. The two- and three-way combinations of the three antibiotics decreased *E. caudatum* growth after 48 h incubation. All the treatments still had living bacteria after 48 h incubation: 10–30 CFU/ml of cultures that received the growth factors and 110–250 CFU/ml of cultures that received no growth factors.

**Figure 9:**
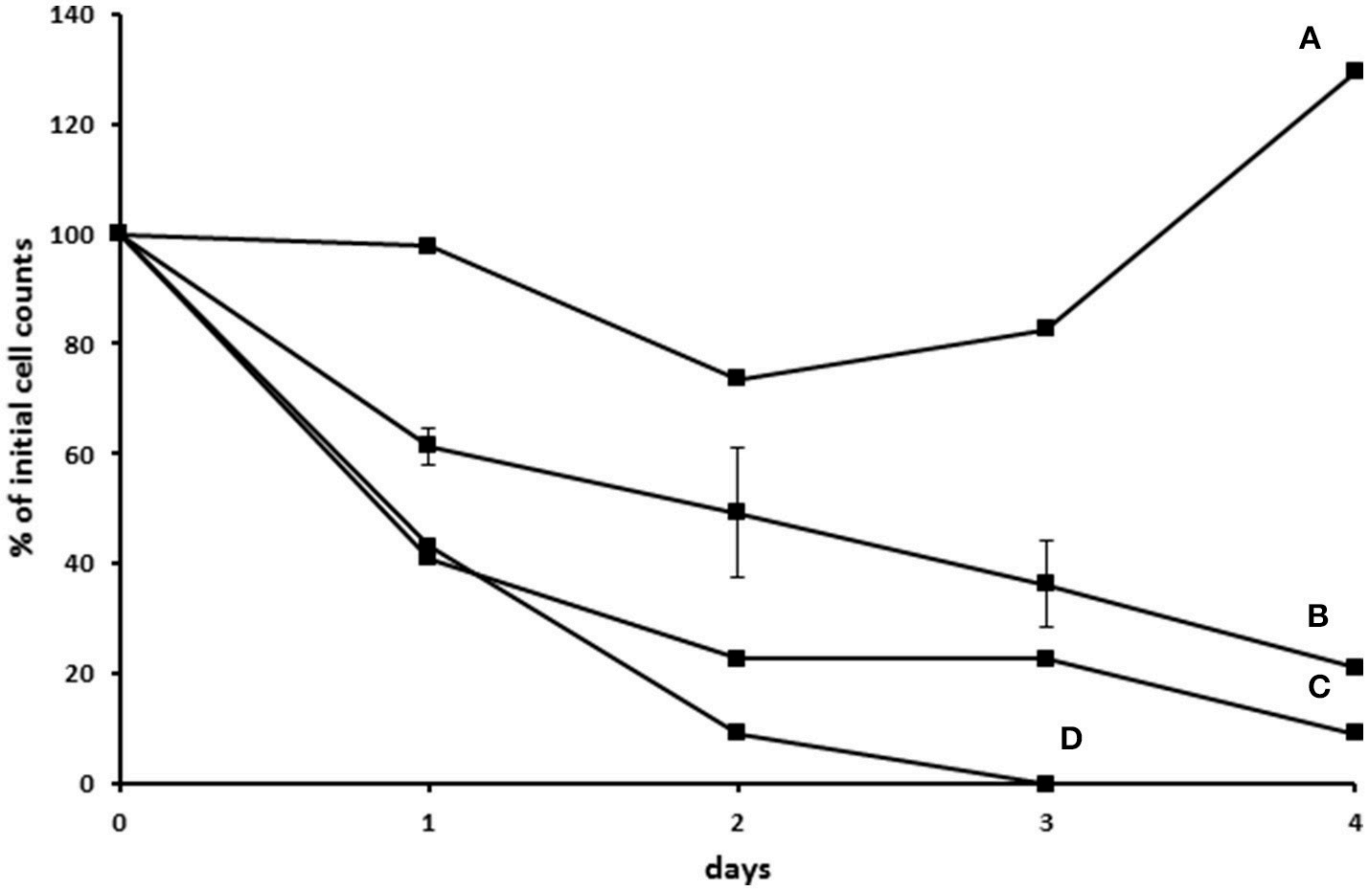
Effect of adding live bacteria (isolated from the *E. caudatum* culture) to the growth of temporarily axenic cultures of *E. caudatum*. **(A)** daily feeding with the mixed bacterial fraction from the culture of *E. caudatum* (not treated with any antibiotics); **(B)** daily feeding with single bacterial isolates from a washed non-antibiotic treated *E. caudatum* culture (average of feeding three bacterial isolates); **(C)** daily feeding of a combination of three bacteria isolated from a washed non-antibiotic treated *E. caudatum* culture; **(D)** control without daily feeding of any live bacteria.

In another attempt to establish an axenic *E. caudatum* culture, we tested a combination of ampicillin (1.0 mg/ml), carbenicillin (1.0 mg/ml), streptomycin (0.2 mg/ml), and oxytetracycline (0.2 mg/ml). After 3 days incubation, no bacterial colonies were seen on TSA plates that had been plated with the antibiotic-treated *E. caudatum* culture directly (without any dilution) and anaerobically incubated for 3 days. After transfer to fresh SP medium containing no antibiotics, however, protozoal cell count gradually declined and became undetectable by light microscopy within 4–5 days (Figure S2). Daily addition of fetal bovine serum, stigmasterol, hemin, and bovine serum albumin, both alone and combinations thereof, to the temporarily axenic *E. caudatum* culture failed to stop the protozoal cell count decline (Figure S2). When each of the nine individual bacteria isolated from the original *E. caudatum* culture was added to the temporarily axenic *E. caudatum* culture, either individually or in combinations of three thereof, the gradual *E. caudatum* cell count decrease was slowed but continued (Figure 9). When the mixed bacteria separated from the *E. caudatum* culture was added daily, the *E. caudatum* cell count did not decrease by day 1 and then gradually recovered and increased by day 4, whereas in the control that received no live bacterial feeding, *E. caudatum* cells disappeared by day 3 (Figure 9).

## Discussion

Axenic cultures of protozoa are needed to fully understand their metabolism and physiology. Repeated attempts to establish an axenic culture of rumen anaerobic ciliate protozoa at best achieved a temporarily axenic culture that could only be maintained for a very short period (Coleman, 1962; Hino and Kametaka, 1977; Bonhomme et al., 1982a,b). In the present study, we evaluated the inhibition of *E. caudatum* growth by eight antibiotics (including three that inhibit cell wall synthesis and five that inhibits protein synthesis) that have been previously used in establishing axenic cultures of other ciliate species. Our results showed that all the antibiotics inhibited the growth of *E. caudatum* even at or below the concentrations used in developing axenic cultures of species of *Paramecium* and *T. thermophila* (Table 2). Except for ampicillin at 48 h, all the antibiotics exhibited inhibition to *E. caudatum* in a linear manner. However, different linearity was apparent for most of the antibiotics over different doses. Even though a comparison of toxicity at the same concentration is logical, varying concentrations of different antibiotics were tested based on literature information. Even at concentrations lower than that of the other antibiotics, chloramphenicol caused extensive destruction of the cell surface of *E. caudatum* cells. This is consistent with its high toxicity (Feder, 1986), and this antibiotic should be excluded from future efforts to generate axenic cultures of ruminal protozoa.

Effects of antibiotics on ruminal ciliate cell structures have not been investigated, although growth inhibition was observed in experiments to establish axenic cultures (Dehority, 2008). In the present study, we examined the surface structures of *E. caudatum* cells treated with eight antibiotics. Chloramphenicol appeared to cause the worse damage, followed by ampicillin. The degree of damage to the cell surface structure is consistent with the extremely low cell counts of *E. caudatum* (0.5 mg/ml ampicillin and 0.005 mg/ml chloramphenicol at 48 h). We further examined the intracellular structures of the *E. caudatum* cells treated with carbenicillin, an inhibitor of peptidoglycan synthesis, which did not show profound damage to the cell surface of *E. caudatum*. We also examined Normocin™, which is a combination of two antibiotics that inhibits bacterial protein and DNA synthesis and one fungicide that has been used in preventing mammalian cell cultures from bacterial and fungal contamination. As shown by TEM, these two antibiotics damaged the structure of chromatin. It is not known how these antibiotics cause structural damage to the *E. caudatum* chromatin, but such damage might contribute to their antibiotics toxicity.

Disruption of membranous structure and accumulation of intracellular granular polysaccharides are believed signs of dying ruminal ciliates (Zeitz et al., 2011). Such morphological changes were seen in the antibiotics-treated *E. caudatum* cells. The outermost part of the *E. caudatum* cells is the glycocalyx (Ergen et al., 2000). Antibiotics-treated cells had loosened appearance of filamentous glycocalyx (Figure 7), which to our knowledge has not been reported from dying protozoa cell. Damaged glycocalyx may affect association with ectosymbionts in ruminal ciliates (Ng et al., 2016) and/or transport of soluble nutrients in unicellular parasitic protozoa (Naderer and McConville, 2008). The TEM showed few intracellular prokaryotic cells inside of the *E. caudatum* cells. One study detected intracellular prokaryotes as endosymbionts through probe hybridization (Lloyd et al., 1996), but some other authors disputed the presence of intracellular prokaryotes including methanogens as true endosymbionts (Coleman and Hall, 1972; Valle et al., 2015). Because of the lack of hydrogenosomes, *E. caudatum* cells probably do not have methanogens as true endosymbionts. More definitive methodologies, including the use of starved *E. caudatum* cells that have depleted engulfed preys, are needed to determine the presence or absence of true endosymbiotic prokaryotes.

All aerobic eukaryotes contain mitochondria, while most anaerobic eukaryotes contain hydrogenosomes or related mitosomes, both of which have evolved from alpha-*Proteobacteria* (Rotte et al., 2000). Consistent with a previous report (Hackstein et al., 2008) and revealed by the TEM, *E. caudatum* cells contain no hydrogenosomes, but they do contain mitosomes. Although evolved from mitochondria, hydrogenosomes, and mitosomes differ in functions. Hydrogenosomes produce H_2_ and ATP, but mitosomes do not. Mitosomes are involved in Fe-S cluster formation (Hackstein and Tielens, 2010). In *E. caudatum* cells, both fermentation and production of H_2_ and ATP probably take pace in the cytosol. Some of the side effects of many antibiotics are attributed to the residual structural similarity of these two organelles to bacteria (Cohen, 2010; Barnhill et al., 2012; Cohen and Saneto, 2012). Even though mitosomes are not involved in energy production, antibiotics can still inhibit *E. caudatum* by inhibiting Fe-S cluster formation in its mitosomes. In addition, many antibiotics, such as aminoglycoside antibiotics (Rizzi and Hirose, 2007), penicillin (Matsuda et al., 2002), and chloramphenicol (Holt et al., 1997), induce apoptotic cell death of mammalian cells, and mitochondria are believed to mediate apoptosis in mammalian cells (Xiong et al., 2014) and aerobic protists (Chose et al., 2003). However, it is not known if mitosomes are also involved in apoptosis. Toxicity to mitosomes and apoptosis, mediated by either mitosomes or another cellular structure of *E. caudatum*, may be further investigated as possible reasons of antibiotic toxicity to *E. caudatum*. The mobility inhibition by chloramphenicol observed in *T. thermophila* (Wu et al., 1996) may not be excluded as a type of toxicity, at least for chloramphenicol. Aminoglycosides at 5 mM (or 2.9 mg/ml for streptomycin), a concentration that is higher than those used in the present study, was not noxious for the growth of *Tetrahymena* spp. (Eustice and Wilhelm, 1984), suggesting that *E. caudatum* might be more sensitive to the toxicity of antibiotics than *Tetrahymena* spp.

The *E. caudatum* cells treated with carbenicillin or Normocin™ showed increased accumulation of glycogen granules. This is consistent with the chemical analysis that showed glycogen is the major carbohydrate reserve in *E. caudatum* (Denton et al., 2015). Repression of glycogen phosphorylase via undetermined mechanism by antibiotics might be one reason of accumulated glycogen granules in the endoplasm of antibiotics-treated *E. caudatum* cells. Inhibited glycogen utilization may also increase accumulation of glycogen granules and contribute to the observed antibiotic toxicity either by lowering ATP availability or by physical obstruction to normal cellular processes. Upon removal of endosymbionts, the resultant aposymbiotic *Euplotes harpa* and *E. aediculatus* also increased accumulation of intracellular glycogen granules (Vannini et al., 2003). It was suggested that the endosymbionts of these two *Euplotes* species produce some metabolites that are needed for glycogenolysis. The same authors also suggested that glycogenolysis provides the energy for *Euplotes* species to grow (Vannini et al., 2007). The results of Vannini et al. and ours indicate that glycogen is an important energy source and the antibiotic treatment might have deprived *E. caudatum* of the ability to produce energy for growth.

Combinations of antibiotics are the primary strategy to develop axenic cultures of ciliates, including attempts to generate axenic cultures of ruminal protozoa (Table 2). β-Lactam antibiotics that inhibit bacterial peptidoglycan synthesis and antibiotics that inhibit prokaryotic protein synthesis are most commonly used in combinations to obtain axenic cultures of unicellular eukaryotes (Allen and Nerad, 1978; Cassidy-Hanley, 2012). A number of non-ruminal ciliates have been successfully cultured and maintained as axenic cultures, but no attempt has succeeded in establishing any axenic ruminal protozoal cultures that can be maintained for repeated use in laboratory research. Although less toxic, carbenicillin, bacitracin, and neomycin failed to eliminate all the bacteria present in the original *E. caudatum* culture at the tested concentrations. The combination of ampicillin (1.0 mg/ml), carbenicillin (1.0 mg/ml), streptomycin (0.2 mg/ml), and oxytetracycline (0.2 mg/ml) successfully eliminated the associated bacteria resulting in a temporarily axenic *E. caudatum* culture lasting for about 3 days. We tested most of the growth factors that have been used in growing axenic cultures of other protozoal species (Allen and Nerad, 1978; Wagener and Pfennig, 1987; Christensen et al., 1996; Mori et al., 2011), both individually or in combinations, but none of them could help the survival of the temporarily axenic *E. caudatum*. Intriguingly, the bacterial fraction reversed the decline of cell counts in the temporary *E. caudatum* culture, whereas the bacteria isolated from the washed *E. caudatum* cells, either individually or in three-way combinations, did not. These observations suggest that certain live bacteria allow *E. caudatum* to repair antibiotics-induced cellular damage and are essential for its long-term survival. The *E. caudatum* cell counts and the OD in the antibiotics-treated *E. caudatum* cultures also appeared to be correlated.

Collectively, these results suggest that *E. caudatum* requires live bacteria of certain species for its survival and growth. It is speculative, but the constant and reliable presence of some ruminal bacteria might have led to the loss of some essential capability to synthesize essential metabolites by *E. caudatum*. This is consistent with the proposed nutritional and metabolic dependence of the ruminal ciliates on their living prey (Fondevila and Dehority, 2001). Antibiotics probably cause both direct cytotoxicity to *E. caudatum* and indirect inhibition by killing the bacteria that are needed for its long-term survival. Cultures of *E. caudatum* with one or more bacteria, instead of axenic cultures, may be developed for future research of its physiology and metabolism. The bacterial fraction of the *E. caudatum* culture can be serially diluted, and each dilution can be tested for its ability to “rescue” the temporarily axenic *E. caudatum* culture. The required bacteria by *E. caudatum* can then be isolated from the dilution that can rescue the temporarily axenic *E. caudatum* culture. Genomic studies of *E. caudatum* may help determine the metabolic potential and physiological requirement and thus help establish axenic cultures of *E. caudatum*.

## Conclusion

*E. caudatum* has few endo- or ecto-symbionts. Antibiotics commonly used to develop axenic cultures of protozoa and to decontaminate mammalian cell cultures inhibited the growth of *E. caudatum*, in an antibiotics- and concentration-dependent manner. Damage to the cell surface and nuclei and increased accumulation of intracellular glycogen granules were evident morphological changes upon antibiotic treatments. A temporarily axenic culture of *E. caudatum* was prepared using combinations of antibiotics, but it could not be maintained. Live bacteria are probably required for the survival and growth of *E. caudatum*. The results of this study can help future research in understanding the relationship between ruminal protozoa and their prokaryotic partners. This study can also be helpful for future effort to develop new strategies to establish monoxenic or polyxenic cultures of *E. caudatum* and other species of ruminal protozoa.

## Author contributions

TP and ZY conceived and designed the experiment. TP executed the experiment and wrote the manuscript. TM contributed to the experiment. JF and ZY revised the manuscript.

### Conflict of interest statement

The authors declare that the research was conducted in the absence of any commercial or financial relationships that could be construed as a potential conflict of interest.
