# Corrigendum: Inhibition of the Rumen Ciliate *Entodinium caudatum* by Antibiotics

**DOI:** 10.3389/fmicb.2017.01504

**Published:** 2017-08-07

**Authors:** Tansol Park, Tea Meulia, Jeffrey L. Firkins, Zhongtang Yu

**Affiliations:** ^1^Department of Animal Sciences, The Ohio State University Columbus, OH, United States; ^2^Molecular and Cellular Imaging Center, Ohio Agricultural Research and Development Center and the Department of Plant Pathology, Ohio State University Wooster, OH, United States

**Keywords:** antibiotics, associated bacteria, axenic culture, *Entodinium*, ruminal protozoa

In the original article, there was an error in the title of **Table 3** as published. ***E. caudatum*** **data (% of that of the control) in the presence of different antibiotics (mg/ml)**. The correct legend appears below.

***E. caudatum*** **data (proportion of that of the control) in the presence of different antibiotics (mg/ml)**.

In the original article, there was a typo in the legend for **Figure 8** as published. **heminm**. The correct legend appears below.

**hemin**

In the original article, there was an error. **The axenic cultures of these ciliated can be maintained in laboratory, and they have greatly facilitated or enabled characterization of their metabolism, physiology, and ecology**.

A correction has been made to **INTRODUCTION:**

**The axenic cultures of these ciliates can be maintained in laboratory, and they have greatly facilitated or enabled characterization of their metabolism, physiology, and ecology**.

In the original article, there was an error. **After two changes of the hexamethyldisilazane, the cells air-dried a chemical hood**.

A correction has been made to **MATERIALS AND METHODS**, **Experiment 1: Growth Inhibition of** ***E. caudatum*** **and Its Associated Prokaryotes by Individual Antibiotics, Electron Microscopy, 2: After two changes of the hexamethyldisilazane, the cells were air-dried in a chemical hood**.

In the original article, there was an error. **The** ***E. caudatum*** **counts in the antibiotics-treated cultures were expressed as % of that of the control culture that received no antibiotics, and culture OD (as an estimate of bacterial concentration) was subjected to two-way ANOVA using SAS 9.3 (SAS Institute, Cary, NC, USA)**.

A correction has been made to **MATERIALS AND METHODS**, **Statistical Analysis, 1: The** ***E. caudatum*** **counts in the antibiotics-treated cultures were expressed as proportion of that of the control culture that received no antibiotics, and culture OD (as an estimate of bacterial concentration) was subjected to two-way ANOVA using SAS 9.3 (SAS Institute, Cary, NC, USA)**.

In the original article, there was an error. **In the 72 h** ***E. caudatum*** **cultures containing 0.5 and 1 mg/ml of ampicillin, very few or no moving** ***E. caudatum*** **cells were seen under the microscope, but unexpectedly 2 mg/m ampicillin only lowered** ***E. caudatum*** **count by** >**54%**.

A correction has been made to **RESULTS, Experiment 1. Growth Inhibition of** ***E. caudatum*** **and Its Associated Prokaryotes by Antibiotics, 1: In the 72 h** ***E. caudatum*** **cultures containing 0.5 and 1 mg/ml of ampicillin, very few or no moving** ***E. caudatum*** **cells were seen under the microscope, but unexpectedly 2 mg/ml ampicillin only lowered** ***E. caudatum*** **count by** >**54%**.

In the original article, there was an error. **Based on the results of Experiment 1, three of the antibiotics (carbenicillin, bacitracin, and neomycin) and their two- and three-way concentrations were used to generate an axenic culture of** ***E. caudatum***.

A correction has been made to **RESULTS, Experiment 2: Preparation of a Temporarily Axenic Culture of** ***E. caudatum*** **and Its Growth Recovery, 1: Based on the results of Experiment 1, three of the antibiotics (carbenicillin, bacitracin, and neomycin) and their two- and three-way combinations were used to generate an axenic culture of** ***E. caudatum***.

The authors apologize for these errors and state that this does not change the scientific conclusions of the article in any way.

## Conflict of interest statement

The authors declare that the research was conducted in the absence of any commercial or financial relationships that could be construed as a potential conflict of interest.

