# Supplementary material for: Inhibition of the Rumen Ciliate Entodinium caudatum by Antibiotics
Source: Front Microbiol. 2017 Jun 28;8:1189. doi: 10.3389/fmicb.2017.01189 (PMC5487518; doi:10.3389/fmicb.2017.01189)
Supplement: Supplementary file 1 [file DataSheet1.docx]

Supplementary Materials

**Inhibition of the rumen ciliate *Entodinium caudatum* by antibiotics**

Running title: Antibiotic inhibition to *Entodinium caudatum* culture

Tansol Park^1^, Tea Meulia^2^, Jeffrey Firkins^1^, and Zhongtang Yu^1,*^

^1^Department of Animal Sciences, The Ohio State University, Columbus, OH, USA;

^2^Molecular and Cellular Imaging Center, Ohio Agricultural Research and Development Center and the Department of Plant Pathology, The Ohio State University, Wooster, OH, USA

*correspondent footnote:

Zhongtang Yu

Department of Animal Sciences

The Ohio State University

2029 Fyffe Road

Columbus, OH 43210

Tel: (614) 292-3057, fax: (614) 292-2929, e-mail: yu.226@osu.edu

# Supplementary Data

**Supplementary Figure S1**. Growth of the prokaryotes in the *E. caudatum* cultures measured as optical density (at 600 nm, Y axis) at different incubation time (hours, X axis). Bars with different letters within the same incubation time differ significantly (n=3).

**Supplementary Figure S2.** The effect of growth factors on the cell counts of temporarily axenic culture of *E*. *caudatum*. All the cultures except the control were grown in fresh SP medium containing soluble starch (0.5%, w/v) as the feed substrate. The temporary axenic *E. caudatum* culture was prepared using a combination of ampicillin (1.0 mg/ml), carbenicillin (1.0 mg/ml), streptomycin (0.2 mg/ml), and oxytetracycline (0.2 mg/ml). The concentration of each growth factor was the same as in Figure 7. GFs includes all the 4 growth factors.

| **Supplementary Table S1**. Number of glycogen granules and intracellular prokaryotes revealed by TEM. | | | | |
| --- | --- | --- | --- | --- |
|  | No. of sections | No. of polysaccharide granules / section | No. of intact prokaryotes  per section | No. of dividing prokaryotes per section |
| Control | 10 | 114^b^ | 1 | 0.6 / section |
| C24 | 5 | 117^b^ | 0 | 0 |
| C48 | 3 | 149^ab^ | 0 | 0 |
| N24 | 3 | 117^b^ | 0 | 0 |
| N48 | 3 | 177^a^ | 0 | 0 |
| C=carbenicillin treated; N=Normocin^TM^ treated. Each antibiotic was treated for 24 h (C24 and N24) or 48 h (C48 and N48). | | | | |
